# Intimate partner violence adversely impacts health over 16 years and across generations: A longitudinal cohort study

**DOI:** 10.1371/journal.pone.0178138

**Published:** 2017-06-05

**Authors:** Deborah Loxton, Xenia Dolja-Gore, Amy E. Anderson, Natalie Townsend

**Affiliations:** Research Centre for Generational Health and Ageing, School of Medicine and Public Health, University of Newcastle, Callaghan, New South Wales, Australia; University of Westminster, UNITED KINGDOM

## Abstract

**Objectives:**

To determine the impact of intimate partner violence on women’s mental and physical health over a 16 year period and across three generations.

**Participants:**

Participants were from the Australian Longitudinal study on Women’s Health, a broadly representative national sample of women comprised of three birth cohorts 1973–78, 1946–51 and 1921–26 who were randomly selected from the Australian Medicare (i.e. national health insurer) database in 1996 to participate in the longitudinal health and wellbeing survey. Since baseline, six waves of survey data have been collected. Women from each cohort who had returned all six surveys and had a baseline measure (Survey 1) for intimate partner violence were eligible for the current study.

**Main outcome measures:**

The main outcome of interest was women’s physical and mental health, measured using the Medical Outcome Study Short-Form (SF-36). The experience of intimate partner violence was measured using the survey item ‘Have you ever been in a violent relationship with a partner/spouse?’ Sociodemographic information was also collected.

**Results:**

For all cohorts, women who had lived with intimate partner violence were more likely to report poorer mental health, physical function and general health, and higher levels of bodily pain. Some generational differences existed. Younger women showed a reduction in health associated with the onset of intimate partner violence, which was not apparent for women in the older two groups. In addition, the physical health differences between women born 1921–26 who had and had not experienced intimate partner violence tapered off overtime, whereas these differences remained constant for women born 1973–78 and 1946–51.

**Conclusions:**

Despite generational differences, intimate partner violence adversely impacted on mental and physical health over the 16 year study period and across generations.

## Introduction

Intimate partner violence (IPV) is experienced by an estimated 30% of women worldwide [1] and has devastating impacts on the lives of women who have this experience. IPV can lead to poor mental health [2–4], including depression [5–8], anxiety [5, 9–11], suicidal ideation and suicide attempts [4, 12, 13] and post-traumatic stress disorder [10, 14]. In addition, experiences of IPV are associated with poor self-rated health [2, 3, 15], as well as a higher prevalence of physical health problems [2, 15], chronic pain [2, 4, 15], chronic disease [16] and problems with physical functioning [2, 4]. A number of physical health conditions have been shown to be associated with experiences of IPV, including fatigue, bronchitis, breathing problems, low iron, poor eyesight and hearing, cervical cancer, allergies, vaginal discharge, asthma, and bowel issues [17].

Women across the adult lifespan are affected by IPV [4, 18]. However, the prevalence of IPV varies between different age groups, with young women experiencing higher rates of IPV than mid-age women, and mid-age women experiencing higher rates of IPV than older women [19]. In addition, there is some suggestion that the impact of experiencing IPV is different across generations. Wilke and Vinton [20] found that women over the age of 45 who had experienced IPV had higher rates of chronic mental and physical health conditions compared to women aged 18 to 29 and those aged 30 to 44. However, the conclusions that can be drawn are limited as the study only included women who had experienced abuse. It is unclear whether the poorer health of the oldest age group is due to a different interaction between abuse and health, or because health generally deteriorates with increasing age. Therefore, there is a need to investigate whether the negative impact of experiencing IPV on physical and mental health persists across time, and whether this relationship varies between different generations of women and in relation to IPV onset.

Few studies have prospectively examined the association between intimate partner violence and health. Ouellet-Morin, Fisher [21] found that IPV was associated with later onset of depression in a sample of mothers of twins and a systematic review of longitudinal studies reported that the association between IPV and depression was bidirectional [22]. However, associations between IPV and physical health across the 16 year study period were not observed in these studies. The objective of the current study was to determine the impact of intimate partner violence on women’s mental and physical health over the 16 year study period and across three generations.

## Method

Participants were from the Australian Longitudinal study on Women’s Health (ALSWH), a broadly representative national sample of women comprised of three birth cohorts 1973–78, 1946–51 and 1921–26 who were randomly selected from the Australian Medicare (i.e. national health insurer) database in 1996 to participate in the longitudinal health and wellbeing survey. Women located in rural areas were deliberately oversampled [23, 24]. Since baseline, six waves of survey data have been collected on an approximately three year rolling basis for each cohort (i.e. Survey 1 to Survey 6). The study follows the same women within each cohort over time. Women from each cohort who had returned all six surveys and had a baseline measure (Survey 1) for IPV were eligible for the current study. Ethics approval was granted from the University of Newcastle and the University of Queensland’s Human Research Ethics Committees.

The experience of IPV was measured using the survey item ‘Have you ever been in a violent relationship with a partner/spouse?’ This question was asked at every survey for women born 1973–78, at Surveys 1, 4, 5 and 6 for women born 1946–51, but was only asked at Survey 1 for women born 1921–26. This variable was used to indicate classify IPV. Women born 1973–78 and those born 1946–51 were defined into one of three categories: 1) if they self-reported ‘yes’ to the IPV question at Survey 1, then they were classified as ‘IPV by 1996’; 2) if they answered ‘no’ at Survey 1 but answered ‘yes’ to the IPV question at any following survey, then they were classified as ‘IPV after 1996’; and if they self-reported ‘no’ at Survey 1 and had not answered ‘yes’ in any subsequent survey, then they were classified as ‘Never IPV’. Women born 1921–26 were only asked the IPV question at Survey 1 and therefore defined into one of two categories: 1) if they self-reported ‘yes’, then they were classified as ‘IPV by 1996’ or 2) if they self-reported ‘no’, then they were classified as ‘Never IPV’.

Sociodemographic characteristics of interest were collected from the 1973–78 cohort, 1946–51 cohort and 1921–26 cohort at the baseline survey (1996) and Survey 6 (2012, 2010 and 2011 respectively). Participants were asked about their educational qualifications (less than Year 12, Year 12 or equivalent and post-school/university), marital status (married/living in a de facto relationship, separated/divorced, widowed/never married), ability to manage on their income (impossible/difficult or easy/not too bad) and area of residence (urban/non-urban living).

The outcome of interest was women’s physical and mental health. The Medical Outcome Study Short-Form (SF-36) [25] measuring health-related quality-of-life has been validated as a reliable tool for use in Australian populations [26]. The 36 self-reported questions which were asked in each survey were used to measure eight different dimensions of health, including physical functioning, social functioning, general health, bodily pain, vitality, role-physical (e.g. role limitations due to physical health), role-emotional (e.g. role limitations due to emotional health) and mental health. Scores range from 0 to 100, with higher scores indicating a better state of health or wellbeing. Scores were calculated for each cohort at all six time-points.

### Data analysis

Pearson’s Chi-square tests of association were used to describe each cohort by defined IPV category according to socio-demographic characteristics and financial status across time. All percentages were weighted by area of residence to account for the oversampling of women from rural areas. SF36 sub-scales for general and mental health, physical functioning and bodily pain means and 95% confidence intervals were graphed to assess statistically significant differences between women in the IPV categories across time for all cohorts. The graphs were also used to visually depict differences between the cohorts. All statistical analyses were conducted using SAS software (version 9.3, SAS Institute Inc., Cary, NC, USA).

## Results

Of the 40,395 women who were surveyed at baseline (1996), 16,761 (42%) were eligible for and included in the current project. This included 4,820 (34%) of the women born 1973–78, 8,373 (61%) of the women born 1946–51 and 3,568 (29%) of the women born 1921–26. Women eligible for the study were slightly more likely to have higher educational qualifications but less likely to find it difficult to manage on their income compared to women not eligible for the study. Eligible women from the 1946–51 and the 1921–26 cohort were more likely to be married/partnered, while women from the 1973–78 cohort were less likely to be married/partnered compared to women not eligible for the study.

Sociodemographic characteristics of women born 1973–78, 1946–51 and 1921–26 at Surveys 1 (1996) to 6 (2012, 2010 and 2011 respectively) were explored (see Table 1). Over time, there was an increase in the proportion of women born 1973–78 who had a post-school qualification and who were partnered. The majority of women from all three cohorts lived in urban areas across the study period. Women born 1921–26 were less likely to be highly educated, partnered, or to find it difficult managing on their income compared with women born 1973–78 and 1946–51. Sociodemographic characteristics of the women born 1946–51 remained fairly stable across time.

**Table 1:** Sociodemographic characteristics of women born 1973–78, 1946–51 and 1921–26 at Survey 1 to Survey 6.

	Survey (%)					
	S1	S2	S3	S4	S5	S6
**Post-Year 12 educational qualifications**						
1973–78 cohort 1946–51 cohort 1921–26 cohort	32.6 40.3 20.3	73.4 - -	78.6 - -	81.1 - -	84.8 - -	84.7 45.2 -
**Difficult to manage on income**						
1973–78 cohort 1946–51 cohort 1921–26 cohort	44.9 37.4 22.6	45.2 38.4 21.9	35.1 33.9 21.9	36.8 25.1 18.0	36.3 21.5 15.5	42.0 33.3 13.7
**Urban living**						
1973–78 cohort 1946–51 cohort 1921–26 cohort	68.4 71.3 67.8	66.4 70.5 66.0	68.2 69.7 68.3	67.8 68.1 68.1	66.4 67.0 67.7	67.1 66.1 68.0
**Married/partnered**						
1973–78 cohort 1946–51 cohort 1921–26 cohort	18.7 94.5 35.0	44.2 93.6 42.1	64.20 93.8 49.2	77.2 93.4 38.2	83.3 92.4 30.3	86.2 90.9 22.5

At baseline, 8% of women born 1973–78 and 12% of women born 1946–51 had experienced IPV. At the end of the study period, a total of 1,232 (26%) women born 1973–78 experienced IPV compared with 1,369 (16%) women born 1946–51. Of the 3,568 women born 1921–26 who were eligible for the study, 184 (5%) reported having experienced IPV by the baseline survey, which was considerably less than the other two cohorts.

**Fig 1:**
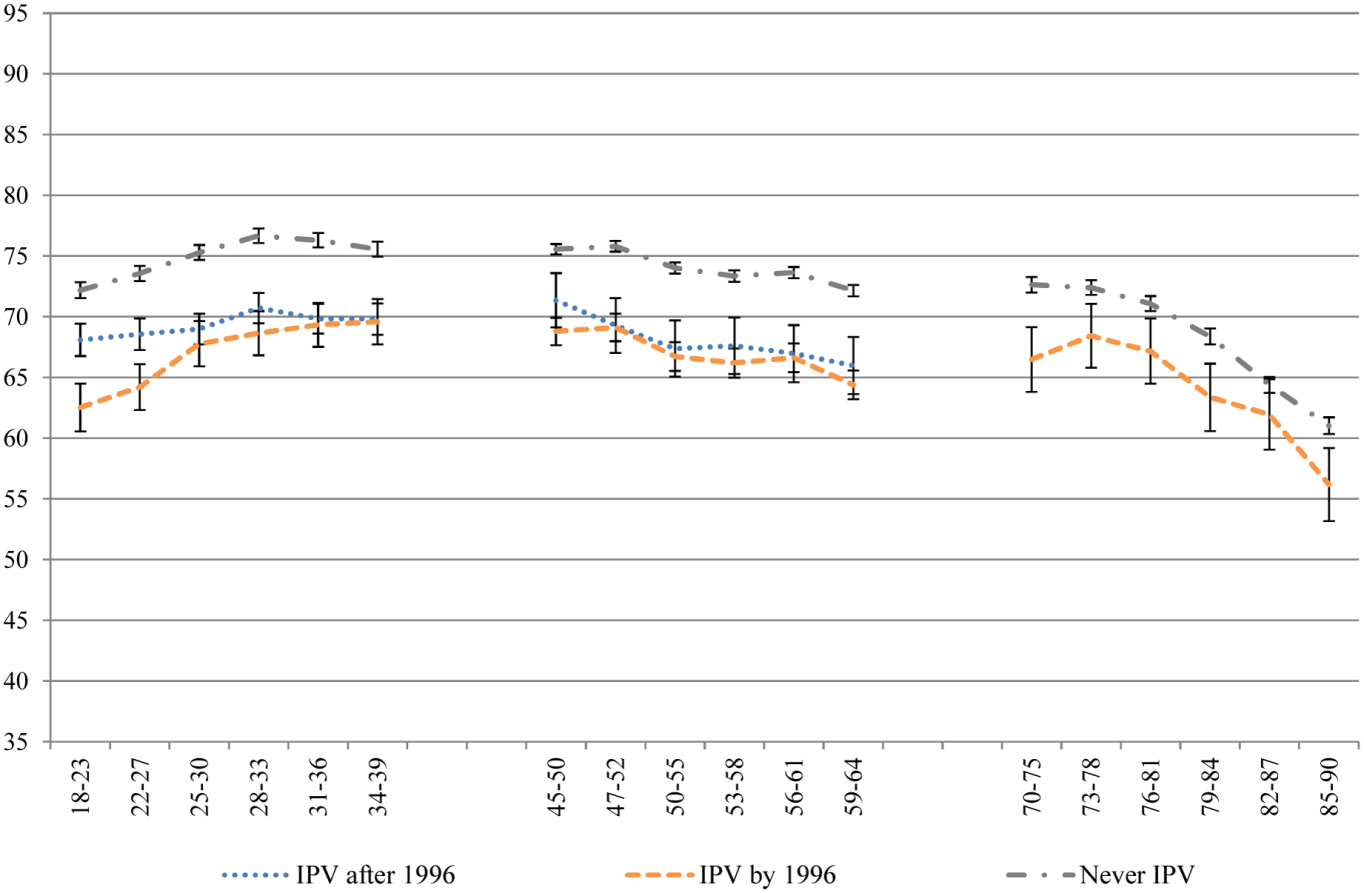
General health of women who had and had not experienced IPV at different time points across the 16 year study period.

**Fig 2:**
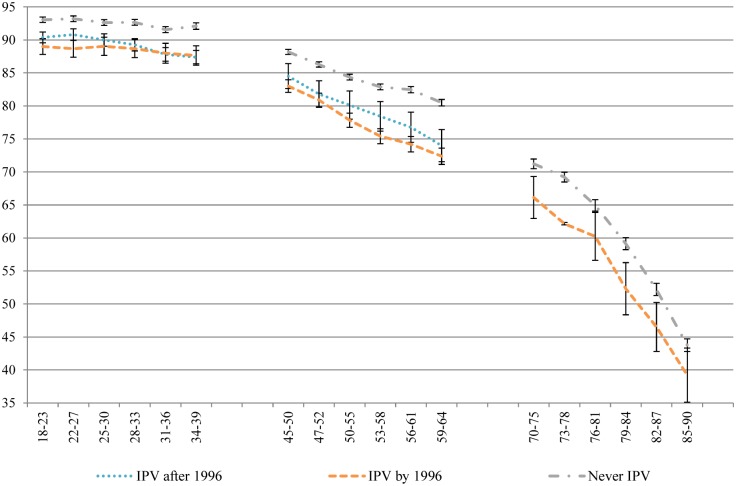
Physical function of women who had and had not experienced IPV at different time points across the 16 year study period.

**Fig 3:**
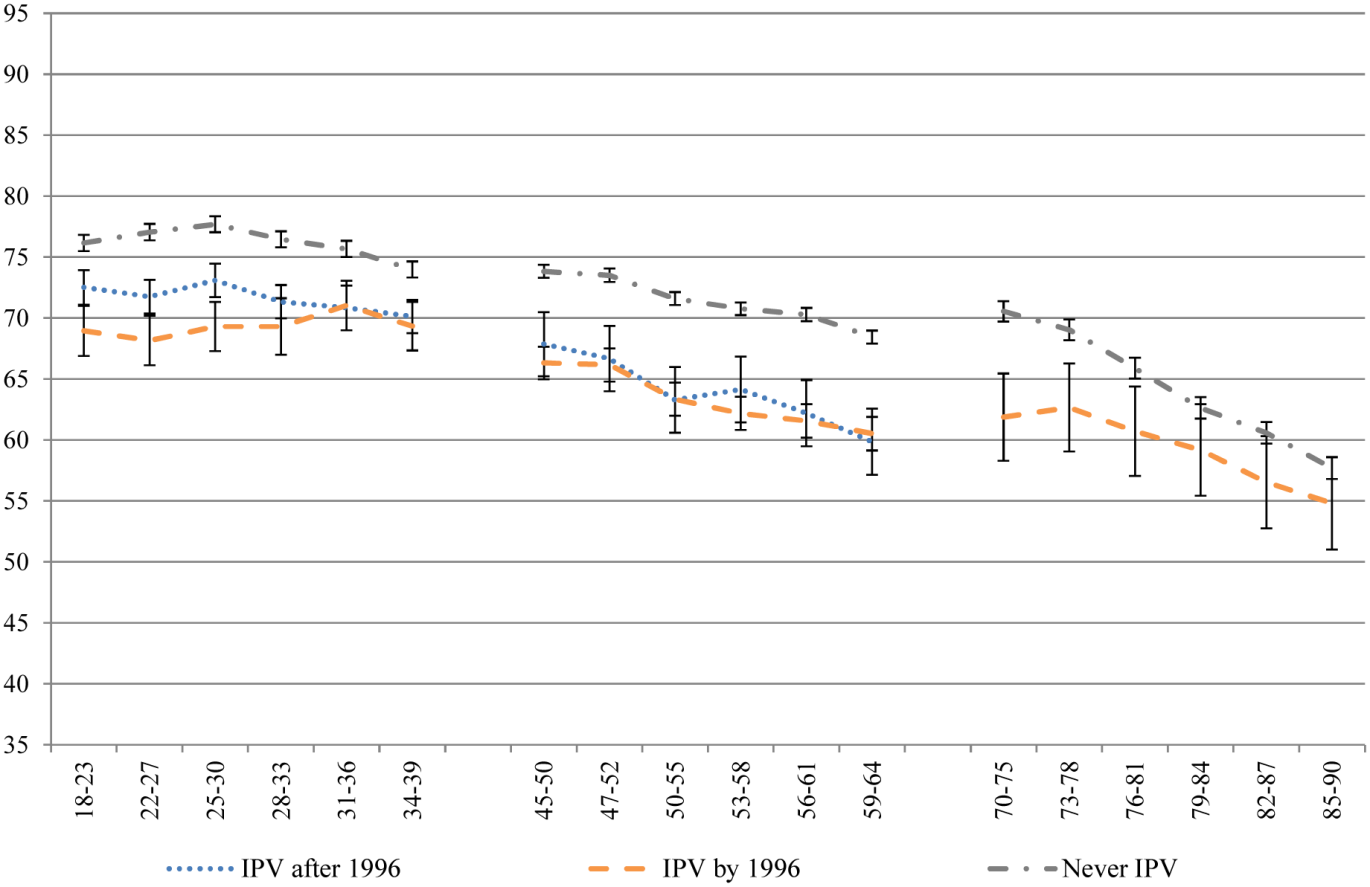
Bodily pain of women who had and had not experienced IPV at different time points across the 16 year study period.

**Fig 4:**
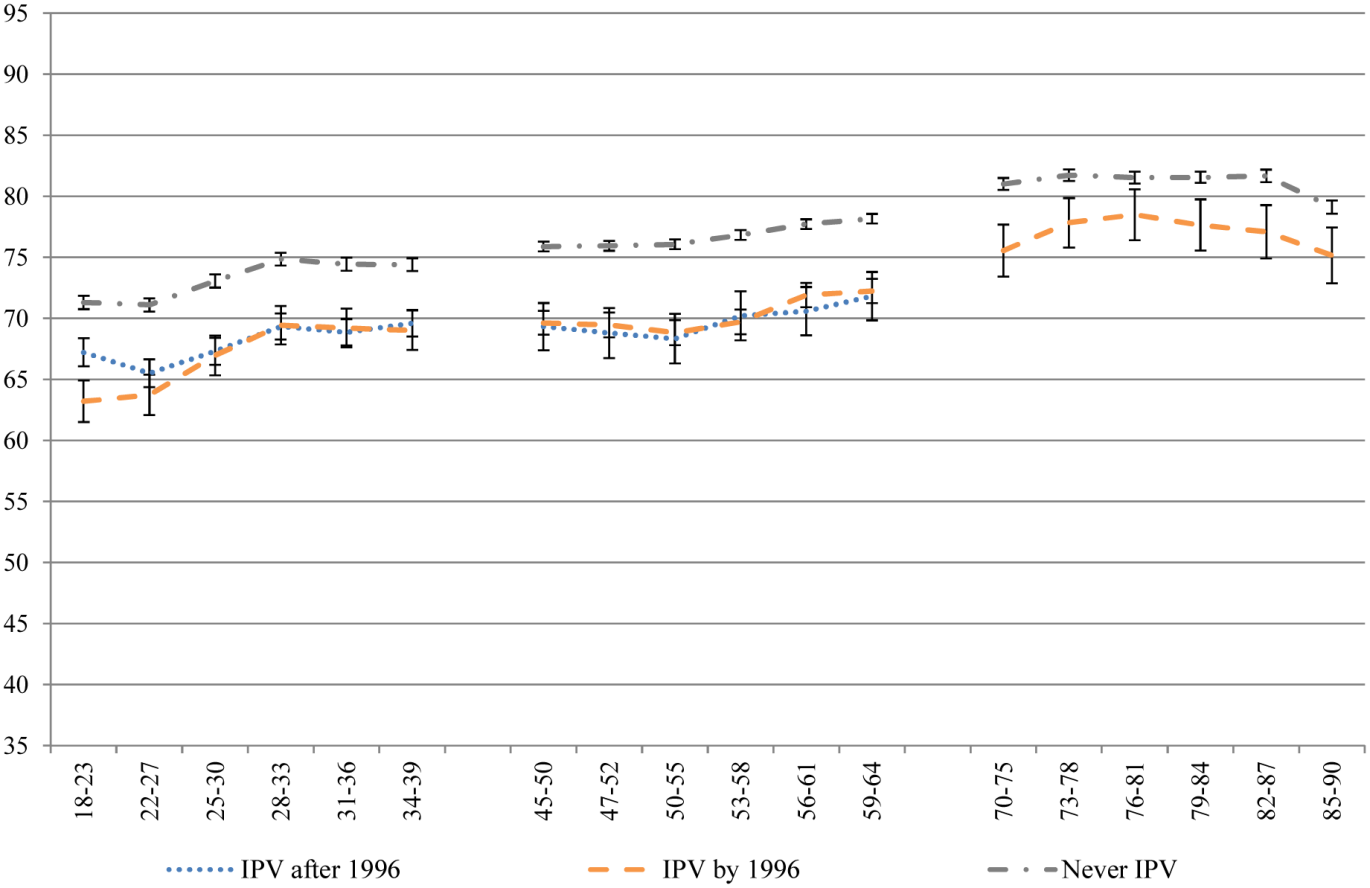
Mental health of women who had and had not experienced IPV at different time points across the 16 year study period.

Despite the use of a conservative, single-item measure of IPV, the results are striking. Across all of the health domains measured by the SF-36, women who had experienced IPV recorded significantly poorer health than women who never experienced IPV, across generations and along the life course. Figs 1–4 contain four subscale scores of the SF-36 for the three cohorts based on IPV experience.

Differences in the trajectories of health were noted between those who had already experienced IPV at baseline and those who went on to experience IPV during the course of the study, and between the generations. Women born 1973–78 who went on to experience IPV during the study had worse health at baseline than women who never experienced IPV, and better health than women who had already experienced IPV at baseline, across all of the health measures. Over time, the scores of women who experienced the onset of IPV during the study merged with those of women who had already experienced IPV at baseline. Although this pattern was apparent for women born 1946–51 on measures of bodily pain, role physical and general health, these associations were not significant. Overall, there were few differences between the two groups who had experienced IPV in the 1946–51 cohort. As women born 1921–26 aged, the differences tapered off between those who had experienced IPV and those who had not.

Physical health measures (general health, physical function, bodily pain and role physical) tended to decline with age, after a small increase in general health among women aged in their late teens and early twenties (Fig 1). Women who experienced IPV had consistently poorer physical health on these four measures (Figs 1–4) than women who never experienced IPV from the ages of 18 to 39; from the ages of 45 to 64; and from 70 until 85. Once women were aged 85, the only significant difference observed for physical health between women who had and had not experienced IPV was for general health.

Women’s reported mental health improved across the 16 year study period, regardless of IPV experience (Fig 4). Those having experienced IPV had poorer mental health overall compared to those who had not experienced IPV, but their mental health similarly improved across the 16 year study period.

## Discussion

This is the first study to demonstrate that the association between intimate partner violence and health outcomes persists over a 16 year period. With the exception of the results for women in their late eighties, women who had experienced IPV reported poorer mental and physical health throughout their lives. Furthermore, differences in health between those who had already experienced IPV at the start of the study and those who first reported IPV after the study had commenced were found for women born 1973–78. However, this was not found for women born 1946–51 or 1921–26.

Results for physical health are strongly suggestive of a lifetime deficit in physical health that is associated with IPV. These results build on past research that has demonstrated a wide range of physical health problems associated with IPV [2–4, 15] by showing that these health issues are persistent. Only the women aged over 85 who had experienced IPV were indistinguishable from their age peers who had never experienced IPV on measures of physical health. However, the prevalence of IPV was low in this older group, possibly reflecting a generational difference in the willingness to disclose IPV or the perception of what defines IPV or the results could be reflective of this small sample size.

Results for mental health were similar, with significant associations between IPV and mental health measures evident across all three cohorts and the 16 year study period. Unlike for physical health, this deficit in mental health was apparent into very old age. This is in keeping with past cross sectional research that has consistently demonstrated this relationship [2] and with longitudinal research that has demonstrated the onset of depression subsequent to IPV [1] and the potential for poorer mental health to both precede and occur subsequent to IPV [27].

The results from the youngest cohort infer that women who have poorer physical and mental health in their late teens and early twenties are at risk of experiencing IPV at a later date. There is a need for more research to look at what happens prior to young adulthood as the results suggest the occurrence of earlier abuse or adversity. Poorer health before the reported IPV in the mid age women underscores the need for greater understanding of what precedes the report of violence. Some potential generational differences were observed in the relationship between IPV and health outcomes over time. Firstly, for women born 1973–78, those who experienced IPV by the baseline survey were worse off than the women that did not experience IPV until after baseline. This was not the case for women born 1946–51 and 1921–26. Secondly, within the 1921–26 cohort, the physical health differences between the women who had and had not experienced IPV tapered off as they aged, whereas these differences remained consistent across time for women born 1973–78 and 1946–51.

As with all research, this study has some limitations. The results in this manuscript are descriptive, and the measures have not been adjusted for possible confounding factors. However, the results are based on a large nationally representative longitudinal sample, which has shown statistically significant differences over a 16 year period between women who have and have not experienced IPV and that have not previously been reported in the literature. The measure of IPV was also conservative and did not take into account different types of abuse or abusive acts, or the identity of the perpetrator, which may have resulted in an underestimation of IPV. The current study sought to examine the health impact on women of living in a violent relationship and the available data were able to answer the research question. There were differences between those eligible and ineligible for inclusion in the analyses, however, previous studies have shown that relationships between variables in the ALSWH remain valid despite missing cases and missing data [28, 29]. Gaps in the literature exist based on the lack of studies able to address both longitudinal and comparative generational analysis on IPV [3, 30]. This study has been able to add to the literature by exploring the health and well-being outcomes by generations over time. Women across generations experiencing IPV have been adversely affected resulting in generally poorer health extending across their lifetimes.
